# Comparisons of liver transplant from DCD outcomes in high-utilization centers versus low-utilization centers in the US: a systematic review and meta-analysis

**DOI:** 10.3389/fimmu.2025.1564551

**Published:** 2025-05-09

**Authors:** Joao Lucas Lima Manzi, Eduarda Saba Oliveira, Sarah Rombach, Vitor Turra, Simone Zaragoza, Yanik Bababekov, Trevor Nydam, Alfred Joseph Tector, Rodrigo Vianna, Phillipe Abreu

**Affiliations:** ^1^ Miami Transplant Institute, Jackson Memorial Hospital, University of Miami, Miami, FL, United States; ^2^ Division of Transplant Surgery, Department of Surgery, University of Colorado Anschutz Medical Campus, Denver, CO, United States

**Keywords:** liver transplant, donor after cardiac death, liver transplantation, organ donation, rapid recovery DCD, center volume

## Abstract

**Purpose/Objective:**

Donation after Cardiac Death (DCD) grafts are considered to be inferiors compared to Donation after Brain Death (DBD) grafts. Consequently, many transplant centers employ highly selective criteria for utilizing DCD, while others use them more liberally, potentially gaining greater expertise with this procedure. This disparity in approach raises questions about the impact of organ selection versus the benefits of increased experience with DCD organs. We performed a systematic review and meta-analysis to evaluate various outcomes in centers with high and low utilization rates of DCD.

**Materials/Methods:**

Electronic databases PubMed, Embase, and Cochrane Library were systematically searched up to May 24th, 2023, for studies related to liver transplantation (LT). This search was conducted in accordance with PRISMA guidelines. The inclusion criteria focused on studies involving controlled DCD published within the last five years, and reporting on at least one of the outcomes of interest. Data was extracted and analyzed using a random-effects generalized linear mixed model with a 95% confidence interval (CI). The primary outcomes assessed were patient survival and graft survival. Heterogeneity among the included studies was evaluated using the I2 test, with I2>40% considered significant, and further explored through influence analysis. Subgroup meta-analysis by DCD utilization rate was done for each outcome. An analysis of the Organ Procurement & Transplantation Network (OPTN) database was performed on October 30th to determine the DCD rates and percentiles.

**Results:**

Seven studies encompassing 898 patients were analyzed. In parallel, data from 151 centers were examined using the OPTN database, determining the rates of DCD utilization in each center over the past five years. Centers from the seven studies were divided into five high-utilization centers (HUC) and two low-utilization centers (LUC), with the 80th percentile (13.33% DCDs/Total LT) used as the threshold for high-utilization. Overall, the 1-year patient survival rate was 94.5% (95%CI: 92.4-96.1; I2 = 0%). HUCs had a rate of 94.6% (95%CI: 92.4-96.1; I2 = 0%), and LUCs had 93.7% (95%CI: 79.1-99.2; I2 = 0%), with a p=0.84. The overall 1-year graft survival rate was 90.6% (95% CI: 88.4-92.4; I2 = 0%). HUCs showed a 1-year graft survival of 90.9% (95%CI: 88.4-92.9; I2 = 0%), and LUCs showed 89.4% (95%CI: 83.8-93.2; I2 = 0%), p=0.54. The overall incidence of ischemic cholangiopathy was 10.3% (95% CI:7.9-13.3; I2 = 0%). The total rate of primary nonfunction was 1.5% (95% CI: 0.7-3.1%; I2 = 46%).

**Conclusion:**

Our findings indicate no statistical difference in outcomes between high and low-utilization centers for DCD liver transplants, possibly suggesting that the higher selection in low-utilization centers is compensated by a greater experience in high-utilization centers.

## Highlights

**Question:** What are the differences in outcomes between high and low-utilization centers in liver transplants from donors after cardiac death (DCDs)?
**Findings:** In this meta-analysis of 898 liver transplant patients from DCDs, the high utilization centers showed a 1-year graft survival of 90.9% (95% CI: 88.4 - 92.9; I2 = 0%), and low utilization centers showed 89.4% (95% CI: 83.8 - 93.2; I2 = 0%), with a p-value between groups of 0.54. For 1-year patient survival, high utilization centers had a rate of 94.6% (95% CI: 92.4 - 96.1; I2 = 0%), and low utilization centers had 93.7% (95% CI: 79. - 99.23; I2 = 0%), with a p-value between groups of 0.84.
**Meaning:** Although low-utilization centers have a higher selectivity for DCD organs, there is no statistically relevant difference compared to high-utilization centers, which may be caused by a compensatory higher experience in the latter.

## Introduction

The rising discrepancy between the number of end-stage liver disease recipients on the waiting list and the number of suitable organs remains one of the urgent challenges in the transplant field (1–3). Mortality in the waitlist due to the shortage of donated livers is still very significant (4). The overall rejection rate of livers is high, due to the absence of an objective prediction of organ function and outcomes, leaving the acceptance in the hands of several subjective factors (5). While various DCD-specific risk scores have been developed to provide objective assessments of graft quality and predict post-transplant outcomes (6–8), their widespread adoption and consistent application in clinical practice appear to be incomplete (9). Consequently, the absence of a universally accepted and strictly adhered-to objective scoring system likely contributes to the continued reliance on subjective evaluations by transplant centers when deciding whether to accept a liver offer, particularly for DCD grafts. This persistent influence of subjective factors, despite the advancements in risk stratification tools, is underscored by the persistently high overall liver rejection rate, suggesting a gap between the availability of objective data and its consistent integration into decision-making processes (10).

Historically, donation after cardiac death (DCD) livers emerged as a potential alternative to increase donor supply and alleviate death on the waitlist list (2, 11). The main difference between DCD and donation after brain death (DBD) is the increased warm ischemia time (WIT), due to the length of time between cardiac death and organ cooling (12). The controlled DCDs (Maastricht 3) are preferred due to the presence of an estimated and closely monitored WIT, which results in lower ischemic damage and postoperative consequences when compared to uncontrolled DCDs (12). Nevertheless, DCD livers are considered lower-quality organs due to the slightly worse outcomes compared to DBD, resulting in an underuse of potential life-saving organs (1, 2, 13). This results in an even higher rejection rate for DCD livers, even though it has been shown that accepting a DCD liver results in survival advantages compared to waiting for a DBD liver (1, 14–17).

DCD livers are very susceptible to preservation methods of minimization of their detrimental procurement, such as machine *in vivo* and *ex vivo* perfusion (2). The susceptibility of preservation methods to influencing organ quality has led to the increasing adoption of preservation technologies in the U.S. and worldwide in recent years (18–23). Along with the improvement in ischemic cholangiopathy (IC) and biliary complication rates, with some studies showing a decrease in IC from 9.0% to 0% and a 2.8-fold reduction in biliary anastomotic strictures., the landscape of DCD liver transplantation in the U.S. is rapidly evolving. DCD liver utilization is steadily increasing nationwide, and machine perfusion, once a specialized technique, is now entering routine clinical practice in many transplant centers (21).

Along with a challenging, time-sensitive procurement and a complicated postoperative course, these specificities from DCD liver transplant increase the importance of the experience of the surgical team and the entire center involved in the surgery. In the US, the use of DCD livers is concentrated in a few high-volume centers, and most of the country has a very low usage proportion (14, 16) The main reasons for this refusal of usage mainly due to apprehension over clinical outcomes, the increased financial burden, and the challenging logistics involved in the procurement (14). We hypothesized that the higher selectivity in low-utilization centers might be compensated by higher experience in high-utilization centers.

Considering this possibility, we sought to perform a systematic review and meta-analysis with new data evaluating the outcomes of centers with high utilization of controlled Donor after Cardiac Death (DCD) livers compared to centers with low utilization of controlled DCD livers.

## Methods

This systematic review and meta-analysis was performed and reported in accordance with the *Cochrane Handbook for Systematic Reviews of Interventions* and the Preferred Reporting Items for Systematic Reviews and Meta-Analysis guidelines (16, 17).

### Study eligibility criteria

Studies were included according to the following eligibility criteria: (1) Prospective or retrospective studies of Liver Transplantation (LT) using donor after cardiac death (DCD); (2) Exclusively use of controlled, Maastricht III or IV, DCD (cDCD) or with explicit group analysis; (3) Published within the last 5 years (2018-2023); (4) Reporting on at least one outcome of interest; (5) Outcomes from a center in the United States (24).We excluded studies with no efficacy outcomes reported specifically for cDCD LT patients in an American center. If studies had overlapping patient populations, only the most up-to-date study was included.

### Search strategy and data extraction

We systematically searched the PubMed, Cochrane Library, and EmBase databases on May 24, 2023. The search terms employed were: (“Liver transplantation” OR ‘‘liver transplant’’) AND (“DCD” OR “Donation after Cardiac Death” OR “Donation after circulatory death” OR “Nonheart-beating donation” OR “Non-Heart beating” OR “NHBD” OR “Cardiac death donor” OR “Nonheart-beating donors”). References of relevant studies were manually searched for additional studies. Two authors (J.M.; E.S.) independently extracted data from each included study.

### Rates gathering and group classification

In this meta-analysis, we utilized data from the Organ Procurement and Transplantation Network (OPTN), which is a unified national database that compiles and updates comprehensive information regarding organ donation and transplantation in the United States. The OPTN data utilized in our analysis was current up to September 30th, 2023.

Our study included all transplant centers that had performed at least one liver transplant in the past five years. From this cohort, we calculated the percentile of DCD liver transplants in relation to the total liver transplants (including both DCD and donation after brain death (DBD) liver transplants) at each center.

To classify the transplant centers into high and low-utilization categories, we employed the 80th percentile as the cutoff threshold. Centers above this percentile were categorized as high-utilization centers for DCD liver transplants, while those below were categorized as low-utilization centers. This classification allowed for a comparative analysis of the utilization rates of DCD liver transplant techniques across different transplant centers. By using percentiles, this approach provides a better characterization of the transplant teams’ routine practices and experience, rather than relying solely on absolute numbers, which may not fully reflect differences in practices across centers.

### Endpoints and subgroup analyses

The primary outcomes assessed in this meta-analysis were centered around crucial indicators of transplant efficacy and complications. These included 1-year patient survival, 1-year graft survival, early biliary complications, ischemic cholangiopathy, and acute kidney injury. The data for each of these outcomes was obtained directly, as reported in the source studies, without any reclassification or modification of rates. Additionally, data on biliary complications and the definitions of warm ischemia time (WIT), when available, were included in **Table 1** for reference.

**Table 1:** Comprehensive table summarizing key characteristics of each study, including the use of Normothermic Regional Perfusion (NRP), definitions of warm ischemia time and biliary complications, primary non-function rate, one-year graft and patient survival rates, donor/recipient age, MELD scores, and cold ischemia time.

	NRP	Definitions of warm ischemia time	Definition of biliary complication	Primary non-function (PNF) rate	One-year graft survival	Patient survival	Donor/Recipient age	MELD	Cold ischemia time (hours)
**Meier 2022** (25)	no	-	anastomotic and non-anastomotic stricture and bile leak	-	1 year (88.6%) / 5 year (70.0%)	–	donor 31.7 ± 10.3/ recipient 57.5 ± 9.0	23 (12–32)	7.7 ± 2.6
**Mercado 2022 (** 26)	no	withdrawal of life support to cross clamp	-	1,09%		1 year (89,9%) / 3 years (84,5%) / 5 years (79,3%)	-	-	-
**Kubal 2018** (27)	no		ischemic cholangiopathy and biliary anastomotic strictures	-	1 year (85,7%)	-	-	-	-
**Hobeika 2021** (14)	no	withdrawal of life-sustaining therapies (WLST) and ending at the time of initiation of aortic flushing with cold preservation solution	-	-	-	1 year (92,8%) / 3 years (83,2%) / 5 years (76,5%)	donor: 25,5 (21-37)	22 (13-27)	median 5,6 (5-7,6)
**Finotti 2022 (** 28)	no	-	biliary leak and biliary strictures	0,87%	1 year (95,3%)	1 year (90,7%)	-	-	-
**Shimada 2024** (29)	no	-	-	3,10%	-	-	donor: 40 (28-52)	-	-
**Sanchez- Garcia 2022 (** 30)	no	-	-	-	2 year (96,8%)	2 year (100%)	-	-	-

For the subgroup analyses, we utilized the 80th percentile (p80) cutoff previously established for classifying transplant centers into high and low-utilization categories.

### Study quality assessment

We utilized the ROBINS-I (Risk Of Bias In Non-randomized Studies - of Interventions) tool to evaluate the risk of bias in the included studies. This approach allows for a detailed assessment of bias due to confounding, selection of participants, classification of interventions, deviations from intended interventions, missing data, measurement of outcomes, and selection of the reported result. Each domain was judged on a scale ranging from low to serious risk. Furthermore, the GRADE (Grading of Recommendations Assessment, Development, and Evaluation) approach was applied to evaluate the overall quality of evidence, considering the risk of bias within individual studies and across the body of evidence. Two independent authors conducted the evaluations (V.T.; S.A.), with any disagreements settled by consensus.

### Statistical analysis

Pooled treatment effects for single-proportion outcomes were calculated using a random-effects generalized linear mixed model (GLMM) with a 95% confidence interval (CI) (25). Heterogeneity was examined with tau-squared, Higgin’s & Thompson’s I^2^ test, and Cochran Q test statistics. An I^2^ of at least 40% was considered significant for heterogeneity. Heterogeneity was explored through influence analysis using the leave-one-out method and Baujat plots. Subgroup analysis by utilization rate was done in groups with 1 or more studies reporting a given outcome. Subgroup analysis included pooling subgroup outcomes using a random-effects model, and subgroup effects were compared using a Q-test, with a significant difference defined as p-value<0.05. For statistical analysis, R statistical software v.4.2.3 was used (31).

## Results

### Study selection and baseline characteristics

**Figure 1:**
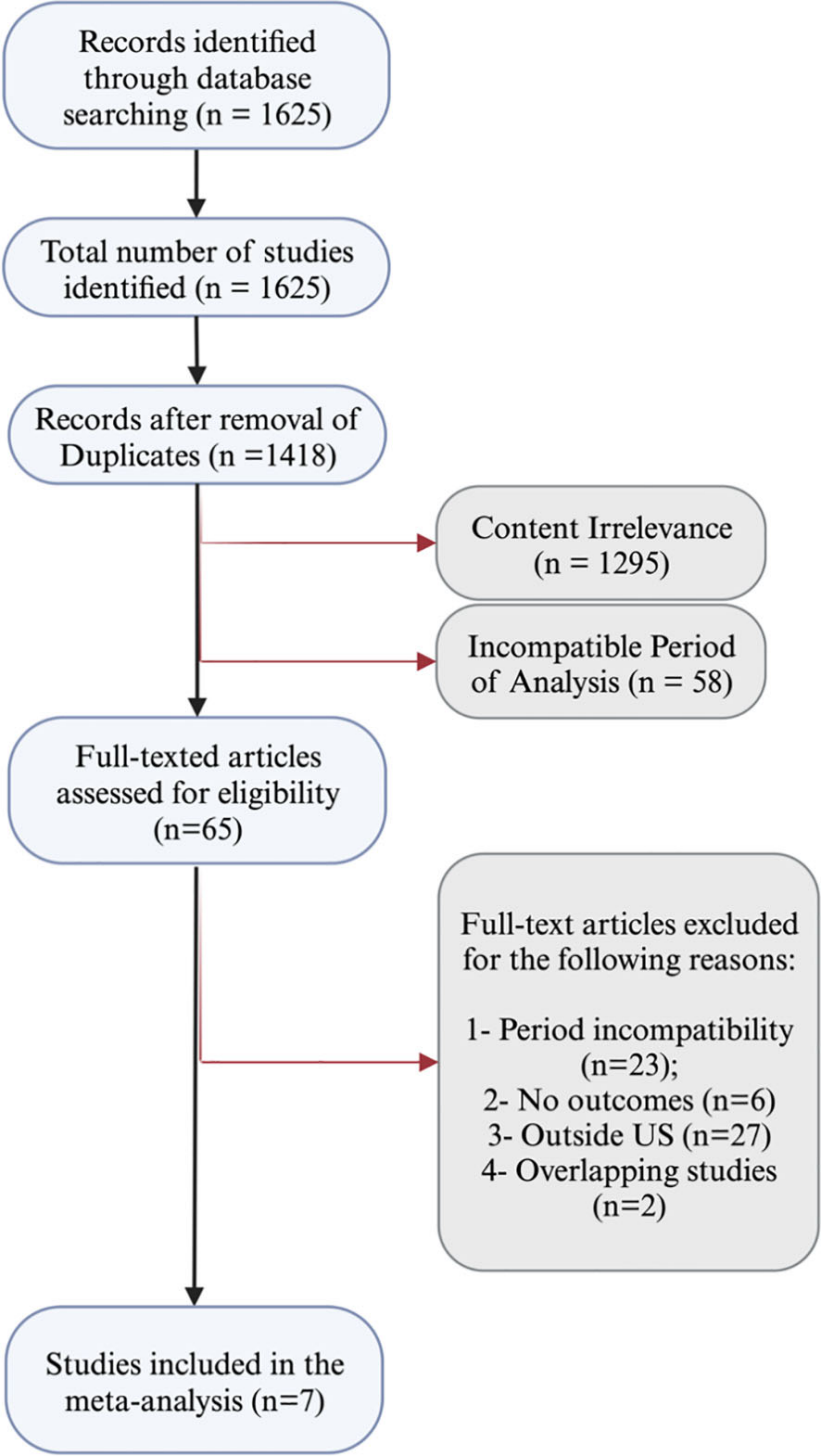
PRISMA flow diagram of study screening and selection.

The initial literature search yielded a total of 1,625 records. After removing 207 duplicates, the pool was narrowed down to 1,418 unique studies. Of these, a majority were excluded based on title and abstract screening, resulting in the exclusion of 1,353 studies. Among these, 1,295 were excluded due to content impertinence, and 58 were excluded based on the period of analysis (**Figure 1**).

### Overall and subgroup outcomes

A total of seven studies encompassing 898 patients were analyzed (25–31). In parallel, data from 152 centers were examined using the OPTN database, determining the rates of DCD (Donation after Cardiac Death) utilization in each center over the past five years (**Supplementary Table**). Centers from the seven studies were divided into five high-utilization and two low-utilization groups, with the 80th percentile (13.3% DCDs relative to the total number of liver transplants in the period) used as the threshold.

**Figure 2:**
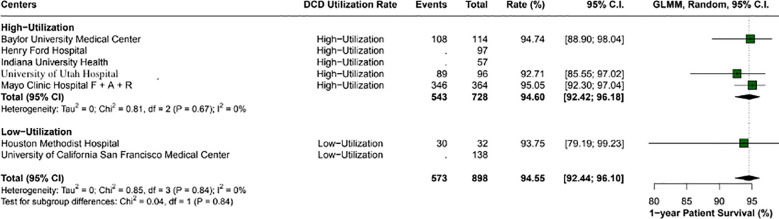
Comparison of 1-year patient survival rates between high and low utilization centers. High utilization centers exhibited a survival rate of 94.6% (95% CI: 92.42 - 96.18), while low utilization centers had a rate of 93.7% (95% CI: 79.19 - 99.23). No significant difference was found between the groups (p-value: 0.84).

**Figure 3:**
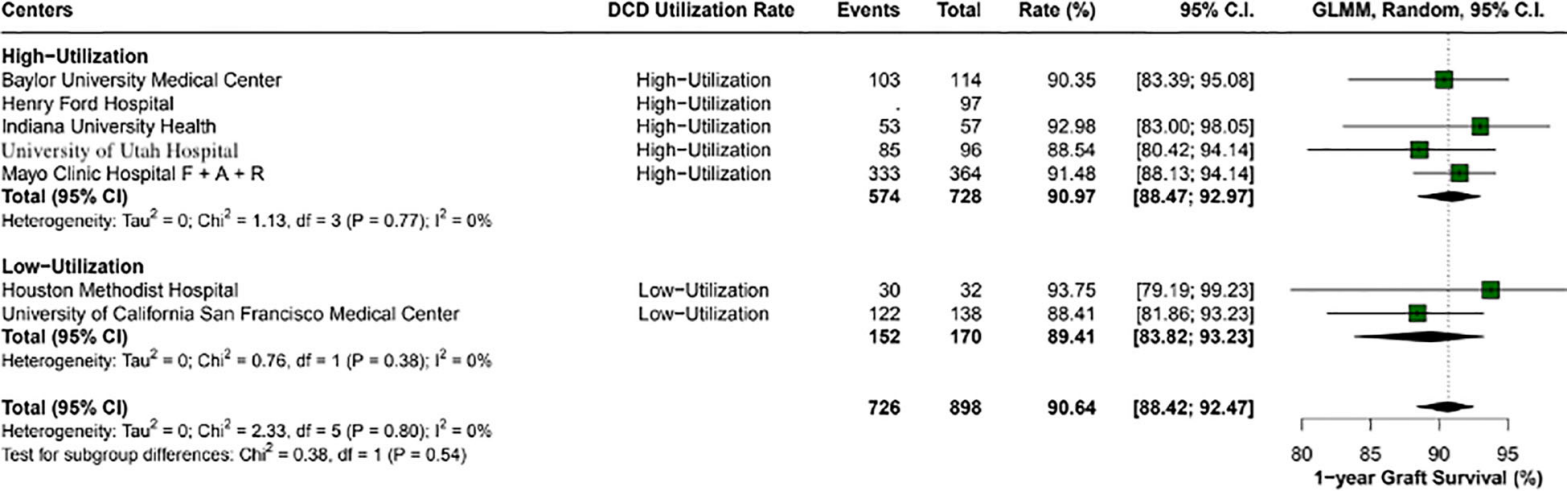
1-year graft survival rates across center utilization categories. High utilization centers had a survival rate of 91% (95% CI: 88.47 - 92.97), compared to 89.4% (95% CI: 83.82 - 93.23) in low utilization centers, with no statistically significant difference observed (p-value: 0.54).

Overall, the 1-year patient survival rate was 94.5% (95% CI: 92.4-96.1; I2 = 0%). High utilization centers had a rate of 94.6% (95% CI: 92.4- 96.1; I2 = 0%), and low utilization centers had 93.7% (95% CI: 79.1 - 99.2; I2 = 0%), with a p-value between groups of 0.84 (**Figure 2**). The 1-year graft survival rate was 90.6% (95% CI: 88.4 - 92.4; I2 = 0%). In comparing high and low utilization centers, high utilization centers showed a 1-year graft survival of 90.9% (95% CI: 88.4 - 92.9; I2 = 0%), and low utilization centers showed 89.4% (95% CI: 83.8 - 93.2; I2 = 0%), with a p-value between groups of 0.54 (**Figure 3**).

**Figure 4:**
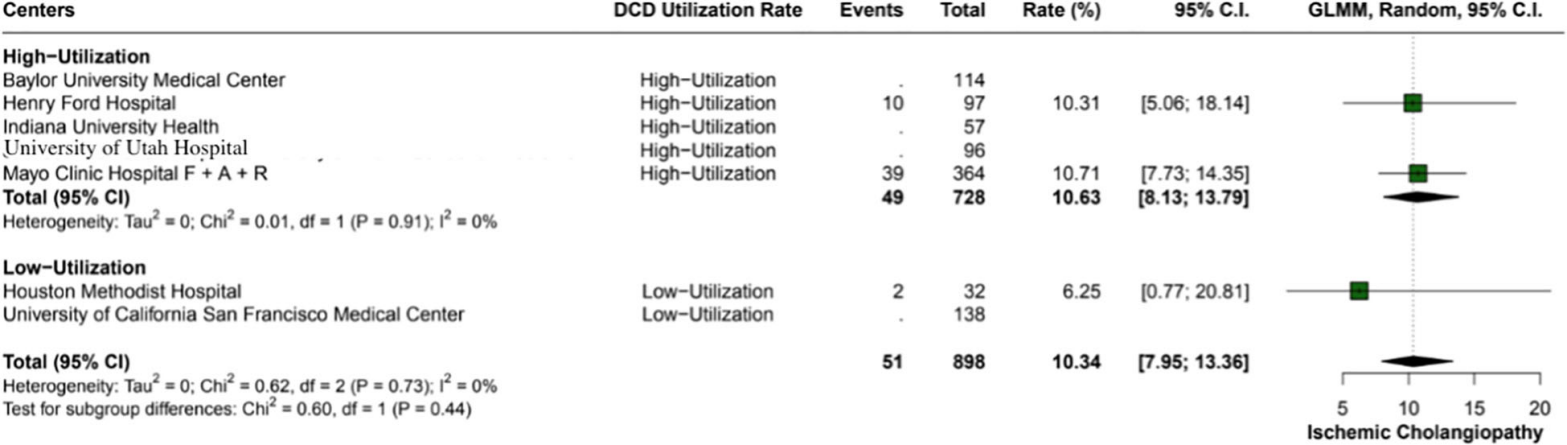
Incidence of ischemic cholangiopathy in the study cohort, observed at an overall rate of 10.3% (95% CI: 7.95 - 13.36). Heterogeneity across studies was non-existent (I2 = 0%).

**Figure 5:**
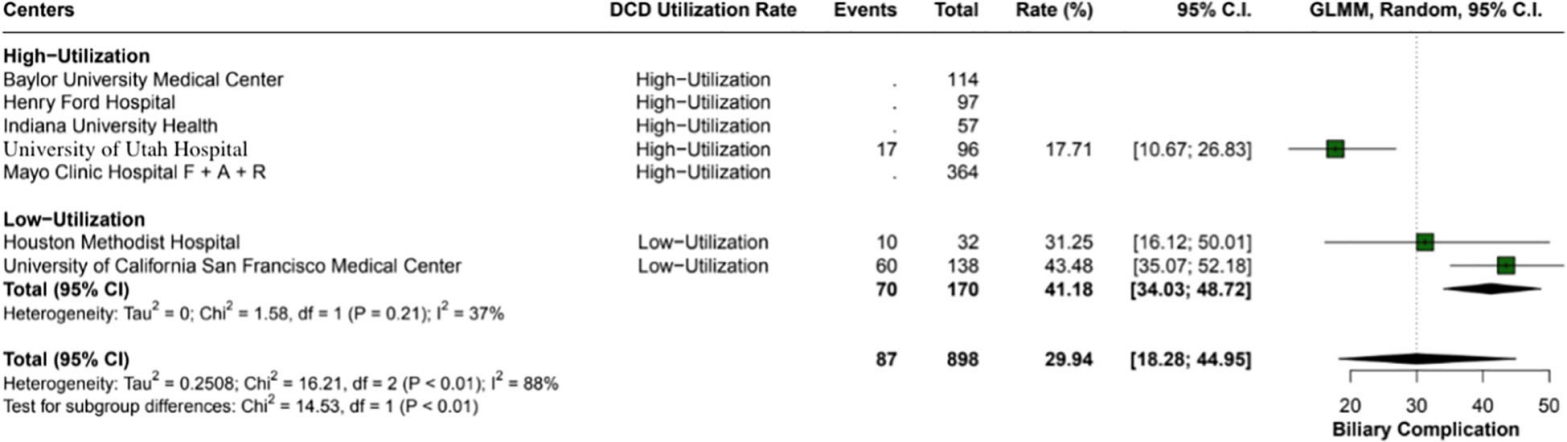
Overall biliary complications in the study, with a total rate of 29.9% (95% CI: 18.28 - 44.95). High utilization centers had a lower incidence rate of 17.7% (95% CI: 18.67 - 26.83) compared to 29.9% (95% CI: 18.28 - 44.95) in low utilization centers, with moderate heterogeneity (I2 = 37%).

**Figure 6:**
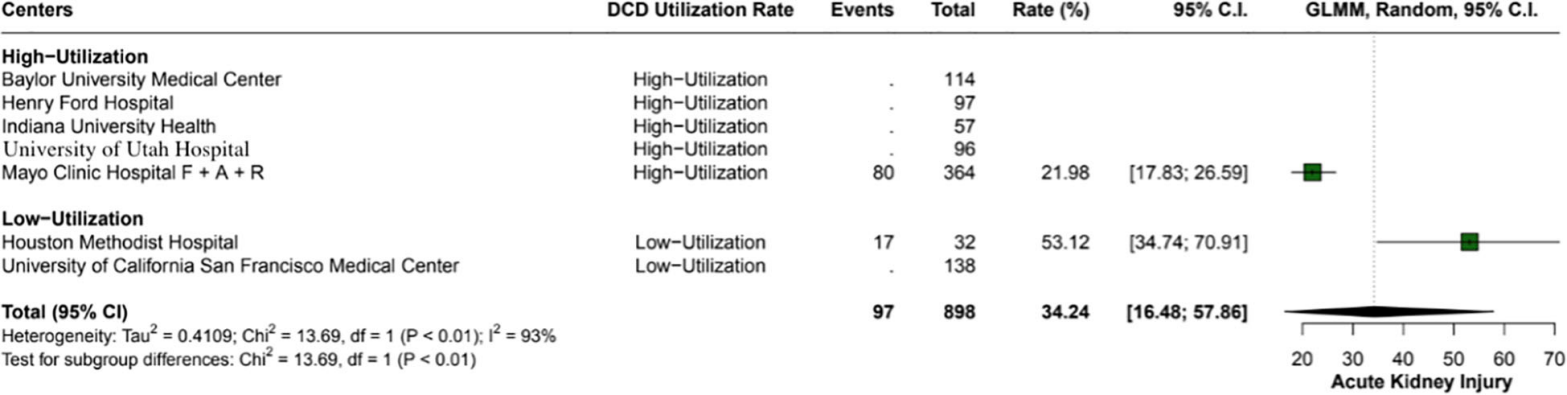
Rates of acute kidney injury in high versus low utilization centers. High utilization centers had a rate of 22% (95% CI: 17.83 - 26.59), while low utilization centers had a significantly higher rate of 53.1% (95% CI: 34.74 - 70.91%), with an overall incidence of 34.2% (95% CI: 16.48 - 57.86%).

**Figure 7:**
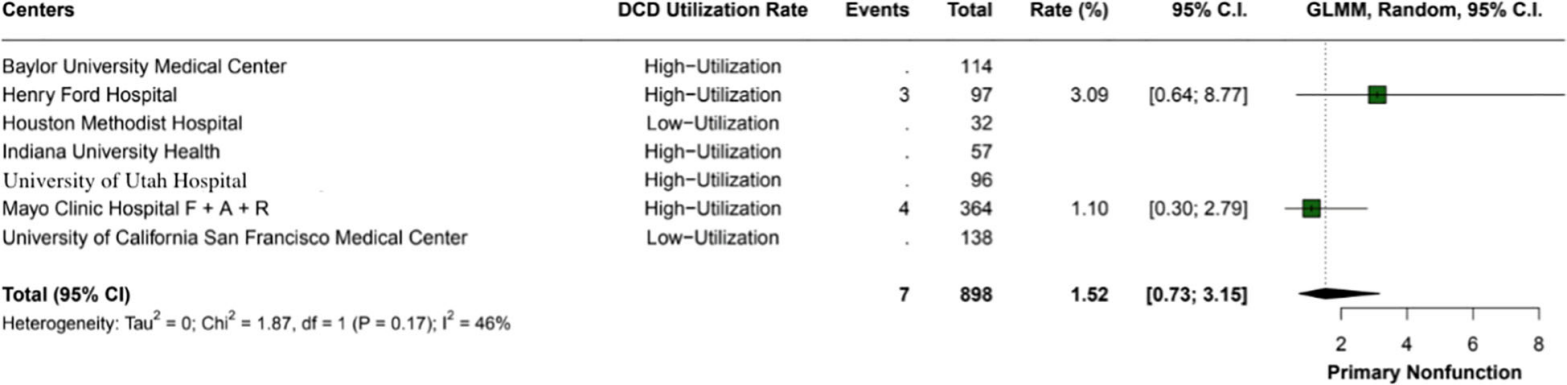
The overall incidence of primary nonfunction within the study was 1.5% (95% CI: 0.73 - 3.15%). The heterogeneity index (I2 = 46%) suggests moderate variability among the included studies.

The overall incidence of ischemic cholangiopathy was 10.3% (95% CI: 7.9 - 13.3; I2 = 0%) (**Figure 4**). Biliary complications occurred at a total rate of 29.9% (95% CI: 18.2 - 44.9; I2 = 88%), with 17.7% (95% CI: 18.6 - 26.8) in high utilization centers and 29.9% (95% CI: 18.2 - 44.9; I2 = 37%) in low utilization centers, p < 0.01 (**Figure 5**). Acute kidney injury was observed in 21.9% (95% CI: 17.8 - 26.5) of cases in high utilization centers and 53.1% (95% CI: 34.7 - 70.9%) in low utilization centers, with an overall incidence of 34.2% (95% CI: 16.4 - 57.8%), p < 0.01 (**Figure 6**). The total rate of primary nonfunction was 1.5% (95% CI: 0.7 - 3.1%; I2 = 46%) (**Figure 7**).

### Study quality assessment

**Figure 8:**
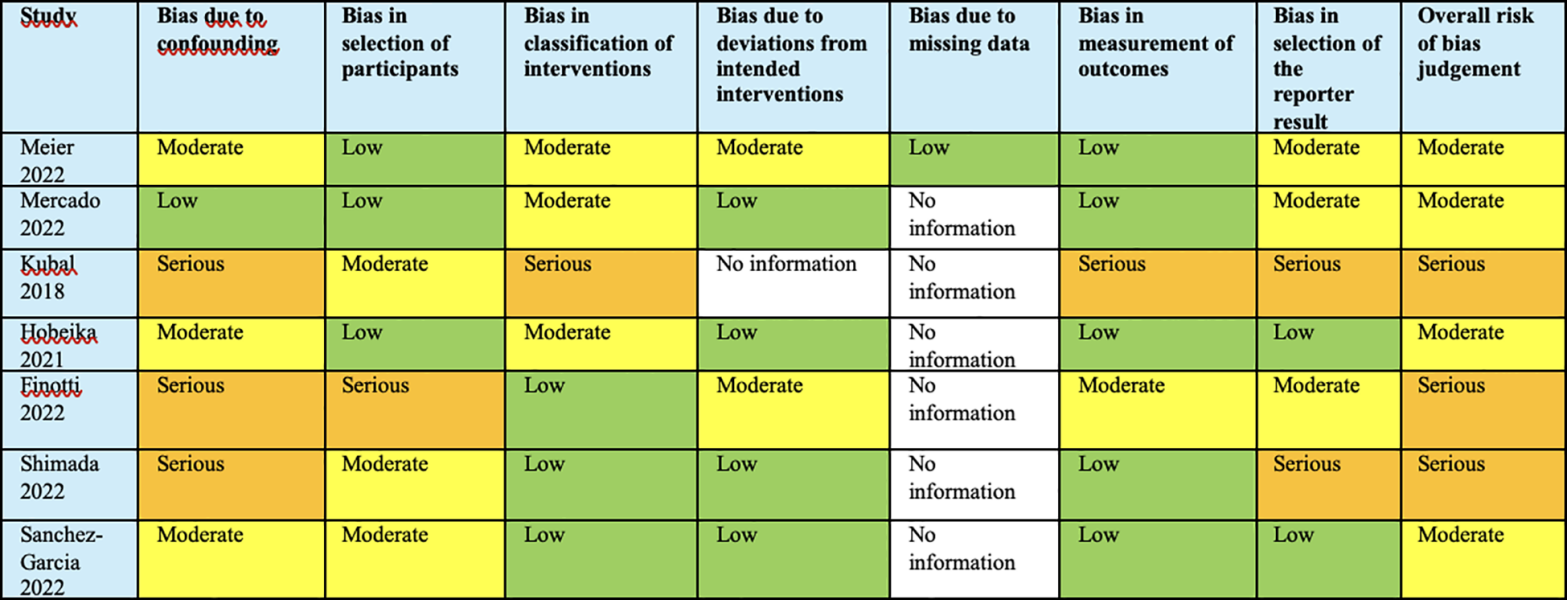
Analysis based on the ROBINS-I and GRADE tools, indicated a nuanced risk of bias across the studies.

Our analysis, based on the ROBINS-I and GRADE tools, indicated a nuanced risk of bias across the studies. Multiple studies demonstrated a moderate risk of bias in various domains, with particular concerns in bias due to confounding and classification of interventions. Serious risks were noted in the studies by Kubal (27), Finotti (28), and Shimada (29), which could significantly affect the validity of the outcomes. The absence of data in several categories across the studies was indicative of an incomplete risk profile. Overall, the evidence quality as assessed is contingent upon these biases, and thus, the strength of our recommendations is calibrated accordingly (**Figure 8**).

## Discussion

This systematic review and meta-analysis of seven studies and 898 patients compared outcomes of centers with high utilization of DCD livers with low utilization of DCD livers. The main findings suggest that (1) there was no difference in 1-year patient survival rate and 1-year graft survival rate between groups.

Traditionally, DCD LT was associated with significantly worse outcomes when compared to DBD LT, specifically higher rates of biliary complication, primary nonfunction, and hepatic artery thrombosis (32–34). These inferior results were likely due to the increased warm ischemia time, poor preservation methods, and rudimentary surgical technique (32, 34). This resulted in widespread reservations regarding the use of DCD LT, which culminated in a smaller dissemination of DCD LT than predicted (35–37). More recently, several studies after the adoption of optimized techniques, better patient selection, protocol standardization, changes in the donor pool, and organ allocation form showed lower rates of complications with higher rates of patient survival (13, 37–40). Croome et al. reported a significant improvement in national outcomes of DCD LT between 2003 and 2014, which was associated with changes in surgical technique, recipient, and donor selection (35). Furthermore, he established a relationship between the gain of experience with DCD grafts and better outcomes stratified by eras (35).

Likewise, it was shown that accepting DCD livers instead of remaining on the waitlist list for a DBD liver provides lower mortality and greater quality-adjusted life years, especially for individuals with more advanced liver disease (36, 41). In accordance with this, Alconchel et al. reported a decrease in 19% of mortality in the waiting list of a single center in Spain after the introduction of controlled DCD liver transplantation in 5 years (17).

In the US, DCD LT is highly concentrated in a few clusters, which is likely due to the residual reservation regarding the worst outcomes related to DCD (14). Hobeika et al. reported that 73,1% of all US liver transplant centers performed at least 1 DCD between 2013-2017 (14). On the other hand, only 3 centers were responsible for 17,5% of all DCD liver transplants in the country, and 11 centers performed nearly half of national DCD LT (46,3%) (14). Regardless of the significant improvement in outcomes of DCD LT, the odds of discard for DCD compared to DBD increased 4,5 times in 2005-2007 or 2015-2017, as reported by Ruck et al. in a US Scientific Registry of Transplant Recipients database logistic regression (38). The concentration of DCD liver transplants in a limited number of centers, coupled with the ongoing reluctance of other centers to adopt DCD livers, exacerbates the disparity in experience and expertise in DCD liver transplantation across the US. As of 2024, DCD utilization is approaching 25–30% (SRTR data), indicating a significant shift in the landscape. As this trend continues, it will be essential to closely monitor long-term outcomes associated with DCD transplants (42, 43).

Our study revealed no difference between the 1-year survival rate and the 1-year graft survival rate between high-utilization and low-utilization centers, reinforcing the rationale that the disadvantages of using the previously called marginal or compromised organs are overcome by the center’s experience regarding organ selection and surgical procedures. In agreement, Ozhathil et al. reported that high-volume utilization centers used higher-risk donor livers, and yet achieved better graft and recipient survival (3) Aligned with this hypothesis, Chau et al. performed an analysis of UNOS Star File between 2016 and 2021, reporting that the top 10% of high-volume DCD centers have significantly improved 6-month and 1-year patient survival when compared to lower-volume centers (44).

Our finding, therefore, mimics a recent analysis by Delman et al. in 2021 that reported that 1-year, long-term survival and graft survival of patients submitted to DCD LT done in centers with more than 5 DCD LT per year are higher than those done in centers with low utilization of DCD LT per year (45). However, his approach was based only on UNOS review, using quantitative data, while this study presents new data from one center and merges literature qualitative data with a meta-analysis.

This meta-analysis has several limitations, the most notable being the retrospective design of most of the included studies. The retrospective nature of these studies inherently introduces selection bias, which could influence the results and their interpretation. Specifically, selection bias may occur due to the non-random selection of patients for donation after circulatory death (DCD), leading to an over- or underestimation of the outcomes. Moreover, unequal prognosis at baseline, such as differences in donor and recipient health status, may contribute to confounding factors that affect the generalizability of the findings.

Furthermore, the lack of patient-level data prevents the stratification of specific subgroups who might benefit more from DCD liver transplants. Without this granular data, it is difficult to identify patient characteristics or clinical factors that predict better outcomes following the use of DCD livers.

An unexpected finding of our meta-analysis was the limited reporting of recipient MELD scores across the included studies, with only two out of the seven studies providing this crucial variable. The MELD score is a well-established predictor of mortality in patients with end-stage liver disease and is frequently used in organ allocation and risk stratification for liver transplantation (9). The absence of this data in a majority of the studies limits our ability to perform more detailed analyses, such as risk-adjusted comparisons or subgroup analyses based on disease severity. Future studies in this area should prioritize the consistent reporting of recipient MELD scores to facilitate more comprehensive evaluations of outcomes in DCD liver transplantation across different center volumes.

Another important limitation is that all the studies included in this meta-analysis are based in the United States. This choice was made intentionally to avoid the significant variability in preoperative practices, surgical procedures, organ optimization protocols, and postoperative care that exist among countries. These differences could introduce substantial heterogeneity and potentially threaten the validity of the results if studies from multiple countries were included. However, this geographical restriction does limit the generalizability of our findings to regions with different transplantation practices. The applicability of our results to countries outside the US, where transplantation standards may differ, remains uncertain. This consideration should be addressed in future studies that explore international variations in outcomes.

This study reflects the pre-machine perfusion era, which constitutes a significant limitation that should be acknowledged. The findings are based on practices and outcomes prior to the widespread adoption of machine perfusion technology. As such, they may not fully capture the potential improvements in graft and patient survival that could arise from the integration of machine perfusion into transplantation protocols. These recent advances have been allowing DCD liver graft to be comparable to DBD grafts. Machine perfusion technology possesses the capacity to significantly enhance organ preservation and improve clinical outcomes, potentially reducing the disparities observed between transplant centers with differing levels of experience and utilization. As the adoption of machine perfusion becomes more widespread, it may create a more equitable landscape, enabling lower-utilization centers to achieve outcomes that are comparable to those of their high-utilization counterparts. This advancement in technology can enhance the viability of marginal organs, rendering them suitable for transplantation and potentially increasing overall graft survival rates. Nevertheless, the sustainability of these findings will likely hinge on the ongoing evolution of clinical practices, training protocols, and the systematic integration of machine perfusion into standard operating procedures across all transplant centers. Consequently, continuous research and data collection will be imperative to monitor these developments and to validate the long-term effects of machine perfusion on both graft and patient survival outcomes.

Despite these limitations, our findings remain hypothesis-generating and may serve as the foundation for future prospective studies with more robust designs, such as cohort studies that incorporate multivariate analysis. This meta-analysis explores the outcomes of the soon-to-end rapid recovery DCD era.

## Conclusion

This systematic review and meta-analysis of nonrandomized studies suggest that the higher selectivity in low-utilization centers might be compensated by the higher experience in high-utilization centers, with no difference in outcomes. These results provide further evidence supporting that high experience with DCD livers has the potential to compensate for the inferior outcomes related to the use of these organs. Prospective trials with multivariate analysis are warranted to confirm this hypothesis.

## Data Availability

The original contributions presented in the study are included in the article/**Supplementary Material**. Further inquiries can be directed to the corresponding author/s.
