# Supplementary material for: Comparisons of liver transplant from DCD outcomes in high-utilization centers versus low-utilization centers in the US: a systematic review and meta-analysis
Source: Front Immunol. 2025 May 9;16:1564551. doi: 10.3389/fimmu.2025.1564551 (PMC12098045; doi:10.3389/fimmu.2025.1564551)
Supplement: Supplementary file 1 [file Table1.pdf]

2018 - 2023 Liver Transplants by center	
**Center name**	**Total**
All Centers	49627
ALCH-TX1 Children's of Alabama	23
ALUA-TX1 University of Alabama Hospital	580
ARUA-TX1 UAMS Medical Center	246
AZCH-TX1 Phoenix Children's Hospital	37
AZGS-TX1 Banner-University Medical Center Phoenix	484
AZMC-TX1 Mayo Clinic Hospital Arizona	1142
AZSJ-TX1 St. Joseph's Hospital and Medical Center	144
AZUA-TX1 Banner University Medical Center-Tucson	59
CACL-TX1 Childrens Hospital Los Angeles	173
CACS-TX1 Cedars-Sinai Medical Center	589
CAGH-TX1 Scripps Green Hospital	155
CALL-TX1 Loma Linda University Medical Center	610
CAMB-TX1 UCSF Medical Center at Mission Bay	53
CAPC-TX1 Lucile Salter Packard Children's Hospital at Stanford	154
CAPM-TX1 California Pacific Medical Center-Van Ness Campus	356
CARC-TX1 Riverside Community Hospital	13
CASD-TX1 University of California San Diego Medical Center	463
CASF-TX1 University of California San Francisco Medical Center	997
CASM-TX1 University of California Davis Medical Center	6
CASU-TX1 Stanford Health Care	577
CASV-TX1 St. Vincent Medical Center	14
CAUC-TX1 University of California at Los Angeles Medical Center	954
CAUH-TX1 Keck Hospital of USC	796
COCH-TX1 Children's Hospital Colorado	86
COPM-TX1 AdventHealth Porter	27
COSL-TX1 Presbyterian/St Luke's Medical Center	111
COUC-TX1 University of Colorado Hospital/Health Science Center	673
CTHH-TX1 Hartford Hospital	161
CTYN-TX1 Yale New Haven Hospital	172
DCGU-TX1 Medstar Georgetown Transplant Institute	696
DCGW-TX1 George Washington University Hospital	30
DEAI-TX1 Nemours Children's Hospital Delaware	33
FLBC-TX1 Broward Health Medical Center	65
FLCC-TX1 Cleveland Clinic Florida Weston	274
FLFH-TX1 AdventHealth Orlando	363
FLJM-TX1 Jackson Memorial Hospital University of Miami School of Medicine	888
FLLM-TX1 Largo Medical Center	97
FLSL-TX1 Mayo Clinic Hospital Florida	887
FLTG-TX1 Tampa General Hospital	834
FLUF-TX1 UF Health Shands Hospital	532
GAEH-TX1 Children's Healthcare of Atlanta at Egleston	130
GAEM-TX1 Emory University Hospital	647
GAPH-TX1 Piedmont Hospital	825

HIQM-TX1 The Queen's Medical Center	86
IAIV-TX1 University of Iowa Hospitals and Clinics Transplant Programs	147
ILCM-TX1 Ann & Robert H. Lurie Children's Hospital of Chicago	74
ILLU-TX1 Loyola University Medical Center	412
ILNM-TX1 Northwestern Memorial Hospital	638
ILPL-TX1 Rush University Medical Center	224
ILUC-TX1 University of Chicago Medical Center	378
ILUI-TX1 University of Illinois Medical Center	316
INIM-TX1 Indiana University Health	922
KSUK-TX1 University of Kansas Hospital	340
KYJH-TX1 Jewish Hospital	118
KYUK-TX1 University of Kentucky Medical Center	345
LACH-TX1 Children's Hospital	2
LAOF-TX1 Ochsner Foundation Hospital	947
LATU-TX1 Tulane Medical Center	102
LAWK-TX1 Willis-Knighton Medical Center	78
MABI-TX1 Beth Israel Deaconess Medical Center	315
MACH-TX1 Boston Children's Hospital	79
MALC-TX1 Lahey Clinic Medical Center	476
MAMG-TX1 Massachusetts General Hospital	479
MANM-TX1 Tufts Medical Center	2
MAUM-TX1 UMass Memorial Medical Center	364
MDJH-TX1 Johns Hopkins Hospital	709
MDUM-TX1 University of Maryland Medical System	568
MIBH-TX1 William Beaumont Hospital	184
MICH-TX1 Children's Hospital of Michigan	11
MIHF-TX1 Henry Ford Hospital	603
MIUM-TX1 University of Michigan Medical Center	492
MNMC-TX1 Mayo Clinic Hospital Minnesota	694
MNSM-TX1 Saint Marys Hospital (Mayo Clinic)	21
MNUM-TX1 University of Minnesota Medical Center, Fairview	574
MOBH-TX1 Barnes-Jewish Hospital	736
MOCG-TX1 Cardinal Glennon Children's Hospital	13
MOCH-TX1 St. Louis Children's Hospital at Washington University Medical Center	91
MOCM-TX1 Children's Mercy Hospital	50
MOLH-TX1 St Luke's Hospital of Kansas City	136
MOSL-TX1 SSM Health Saint Louis University Hospital	183
MSUM-TX1 University of Mississippi Medical Center	264
NCCM-TX1 Carolinas Medical Center	455
NCDU-TX1 Duke University Hospital	700
NCMH-TX1 University of North Carolina Hospitals	208
NEUN-TX1 The Nebraska Medical Center	622
NJLL-TX1 Virtua Our Lady of Lourdes Hospital	100
NJUH-TX1 University Hospital	229
NYCP-TX1 NY Presbyterian Hospital/Columbia Univ. Medical Center	600
NYFL-TX1 Strong Memorial Hospital, University of Rochester Medical Center	400

NYMA-TX1 Montefiore Medical Center	315
NYMS-TX1 Mount Sinai Medical Center	964
NYNS-TX1 North Shore University Hospital/Northwell Health	165
NYNY-TX1 New York-Presbyterian Hospital/Weill Cornell Medical Center	350
NYUC-TX1 NYU Langone Health	372
NYWC-TX1 Westchester Medical Center	348
OHCC-TX1 The Cleveland Clinic Foundation	1086
OHCH-TX1 Nationwide Children's Hospital	18
OHCM-TX1 Children's Hospital Medical Center	146
OHOU-TX1 Ohio State University Medical Center	820
OHUC-TX1 University of Cincinnati Medical Center	810
OHUH-TX1 University Hospitals of Cleveland	114
OKBC-TX1 Integris Baptist Medical Center	446
OKMD-TX1 OU Medical Center	145
ORUO-TX1 Oregon Health and Science University	376
ORVA-TX1 VA Portland Health Care System	107
PAAE-TX1 Albert Einstein Medical Center	378
PAAG-TX1 Allegheny General Hospital	258
PACH-TX1 UPMC Children's Hospital of Pittsburgh	167
PACP-TX1 Children's Hospital of Philadelphia	96
PAGM-TX1 Geisinger Medical Center	74
PAHE-TX1 Penn State Milton S Hershey Medical Center	93
PAHM-TX1 Hahnemann University Hospital	18
PAPT-TX1 University of Pittsburgh Medical Center	737
PARH-TX1 Reading Hospital	3
PATJ-TX1 Thomas Jefferson University Hospital	469
PATU-TX1 Temple University Hospital	50
PAUP-TX1 Hospital of the University of Pennsylvania	834
PAVA-TX1 VA Pittsburgh Healthcare System	177
PRSJ-TX1 Auxilio Mutuo Hospital	205
SCMU-TX1 Medical University of South Carolina	410
SDMK-TX1 Avera McKennan Hospital	48
TNLB-TX1 Le Bonheur Children's Medical Center	18
TNMH-TX1 Methodist University Hospital	604
TNVU-TX1 Vanderbilt University Medical Center	803
TXAS-TX1 Baylor Scott and White All Saints Medical Center-Fort Worth	265
TXBC-TX1 University Hospital, University of Texas Health Science Center	672
TXCM-TX1 Children's Medical Center of Dallas	61
TXHD-TX1 Medical City Dallas Hospital	15
TXHH-TX1 Memorial Hermann Hospital, University of Texas at Houston	177
TXHI-TX1 CHI St. Luke's Health Baylor College of Medicine Medical Center	466
TXHS-TX1 Methodist Specialty and Transplant Hospital	353
TXJS-TX1 University of Texas Medical Branch at Galveston	78
TXMC-TX1 Methodist Dallas Medical Center	403
TXMH-TX1 Houston Methodist Hospital	1063
TXPL-TX1 Medical City Fort Worth	2

TXSP-TX1 UT Southwestern Medical Center/William P. Clements Jr. University Hospital	632
TXTC-TX1 Texas Children's Hospital	215
TXTX-TX1 Baylor University Medical Center	513
TXUC-TX1 University Children's Health	24
TXVA-TX1 Michael E. DeBakey VA Medical Center	58
UTLD-TX1 Intermountain Medical Center	504
UTMC-TX1 University of Utah Medical Center	269
UTPC-TX1 Primary Children's Hospital	69
VAMC-TX1 VCU Health System Authority, VCUMC	782
VAUV-TX1 University of Virginia Health Sciences Center	517
WACH-TX1 Seattle Children's Hospital	81
WASM-TX1 Swedish Medical Center	233
WAUW-TX1 University of Washington Medical Center	657
WICH-TX1 Children's Hospital of Wisconsin	17
WISE-TX1 Froedtert Memorial Lutheran Hospital	160
WISL-TX1 Aurora St. Luke's Medical Center	135
WIUW-TX1 University of Wisconsin Hospital and Clinics	637

**DCD**	**% of DCD livers**
5161	10.399581
0	0
50	8.62068966
67	27.2357724
2	5.40540541
114	23.553719
524	45.8844133
21	14.5833333
8	13.559322
0	0
3	0.50933786
11	7.09677419
49	8.03278689
0	0
0	0
14	3.93258427
0	0
88	19.0064795
128	12.8385156
2	33.3333333
29	5.02599653
0	0
32	3.35429769
7	0.87939698
0	0
2	7.40740741
0	0
113	16.7904903
12	7.45341615
23	13.372093
44	6.32183908
0	0
0	0
0	0
9	3.28467153
12	3.30578512
147	16.5540541
16	16.4948454
200	22.5479143
66	7.91366907
44	8.27067669
0	0
30	4.63678516
58	7.03030303

0	0
20	13.6054422
0	0
75	18.2038835
107	16.7711599
0	0
21	5.55555556
20	6.32911392
141	15.2928417
9	2.64705882
5	4.23728814
36	10.4347826
0	0
137	14.4667371
8	7.84313726
2	2.56410256
55	17.4603175
0	0
0	0
85	17.7453027
0	0
10	2.74725275
44	6.20592384
42	7.3943662
10	5.43478261
0	0
126	20.8955224
73	14.8373984
41	5.90778098
0	0
39	6.79442509
76	10.326087
0	0
0	0
0	0
3	2.20588235
6	3.27868853
36	13.6363636
46	10.1098901
71	10.1428571
10	4.80769231
80	12.8617363
0	0
2	0.87336245
13	2.16666667
32	8

14	4.44444444
121	12.5518672
0	0
9	2.57142857
7	1.88172043
6	1.72413793
153	14.0883978
0	0
0	0
260	31.7073171
36	4.44444444
3	2.63157895
49	10.9865471
2	1.37931035
46	12.2340426
10	9.34579439
3	0.79365079
12	4.65116279
0	0
0	0
1	1.35135135
1	1.07526882
0	0
54	7.32700136
1	33.3333333
27	5.75692964
0	0
17	2.03836931
11	6.21468927
0	0
13	3.17073171
8	16.6666667
0	0
84	13.9072848
51	6.35118306
83	31.3207547
40	5.95238095
0	0
1	6.66666667
0	0
2	0.42918455
0	0
1	1.28205128
53	13.1513648
85	7.99623707
0	0

30	4.74683544
0	0
110	21.4424951
0	0
0	0
106	21.031746
13	4.83271376
0	0
31	3.96419437
36	6.96324952
0	0
14	6.00858369
47	7.15372907
0	0
7	4.375
7	5.18518519
90	14.1287284
